# A Historical Review of Vasoactive Intestinal Peptide and Pituitary Adenylate Cyclase-Activating Polypeptide in Sepsis

**DOI:** 10.3390/biology15090663

**Published:** 2026-04-22

**Authors:** Razia Dawlaty, Philomena Entsie, Emmanuel Boadi Amoafo, Elisabetta Liverani, Glenn P. Dorsam

**Affiliations:** 1 Plant Pathology, Microbiology, and Biotechnology, North Dakota State University, Fargo, ND 58102, USA; razia.dawlaty@ndsu.edu; 2 Departments of Pharmaceutical Sciences, North Dakota State University, Fargo, ND 58102, USA; philomena.entsie@ndsu.edu (P.E.); emmanuel.amoafo@ndsu.edu (E.B.A.); elisabetta.liverani@ndsu.edu (E.L.)

**Keywords:** vasoactive intestinal peptide (VIP), pituitary adenylate cyclase-activating polypeptide (PACAP), sepsis, cytokine storm, neuroimmune regulation, VPAC1, VPAC2, innate immune response, intestinal barrier function, immunomodulation

## Abstract

The neuropeptides vasoactive intestinal peptide (VIP) and pituitary adenylate cyclase-activating polypeptide (PACAP) have emerged as potent modulators of immune responses during sepsis, yet their roles remain complex, alternating between protective and permissive depending on timing, tissue compartment, and inflammatory context. This review presents a historical assessment of VIP and PACAP in sepsis research, highlighting the evolution of conceptual advances across five decades. Starting in the 1980s, early studies revealed that VIP levels rise during endotoxemia and correlated with hypotension and mortality, suggesting a deleterious role. By the 1990s, research pivoted toward understanding gut-derived VIP and its interaction with nitric oxide, culminating in the classification of VIP and PACAP as “macrophage deactivating factors” that downregulate TNFα and IL-6. The 2000s further clarified their cell-specific actions through VPAC1/2 and PAC1 receptors, showing anti-inflammatory effects on both innate and adaptive immune cells, while illuminating delivery challenges overcome by liposomal encapsulation. The 2010s expanded this narrative by dissecting receptor dynamics, gut barrier regulation, and VIP’s role in neuroimmune crosstalk and thrombo-inflammation. Most recently, studies in the 2020s provide a nuanced view of how VIP suppresses inflammatory damage but also enables pathogen persistence during live bacterial infection, implicating VIP signaling in trade-offs between tolerance and clearance. Across this chronological framework, VIP and PACAP have oscillated between friend, foe, and frenemy, underscoring the importance of context in leveraging their therapeutic potential in sepsis.

## Simple Summary

Sepsis is a serious condition in which the body’s response to infection becomes uncontrolled, causing widespread inflammation that can damage organs such as the lungs, kidneys, intestines, and heart. Scientists are studying natural signaling molecules called neuropeptides, including vasoactive intestinal peptide (VIP) and pituitary adenylate cyclase-activating polypeptide (PACAP), because they help regulate communication between the nervous and the immune systems. Over four decades of research has shown that these molecules can reduce harmful inflammation, help restore balance to the immune response, and protect important body tissues. Although their actions are complex and not yet ready to be used directly as treatments, understanding how VIP and PACAP control inflammation may help scientists develop new therapies against sepsis in the future.

## 1. Introduction

Sepsis is a serious and potentially life-threatening medical condition that arises when the host’s immune system responds to infection in a dysregulated manner, causing widespread inflammation and life-threatening organ dysfunction [1]. Sepsis can be caused by a wide range of infections, including bacterial, viral, and fungal infections [2,3,4]. The infection can start anywhere in the body, but commonly starts in the lungs, urinary tract, skin, or abdomen [5,6]. The infection triggers an excessive response of the immune system with the subsequent release of inflammatory molecules, known as the cytokine storm [2]. The overreaction of the immune system leads to organ damage and, if left untreated, can progress to septic shock [2,3,4]. Symptoms of sepsis may include fever, tachycardia, tachypnea, confusion or disorientation, hypotension, decreased urine output, and skin that is warm to the touch [1,6]. Diagnosis of sepsis is based on a combination of symptoms, physical exam, and laboratory tests, such as blood cultures. Treatment for sepsis typically involves antibiotics to target the underlying infection, as well as identifying the source of the infection and removing it [3]. Supportive care to address the symptoms and possible complications are also implemented [3]. In severe cases, hospitalization may be required, and treatment may include intravenous fluids, medications to raise blood pressure, and mechanical ventilation to support breathing [3]. Early recognition and treatment of sepsis is crucial to improving outcomes and reducing the risk of complications and death [3,7]. Vasoactive intestinal peptide (VIP) and its structurally similar peptide, pituitary adenylate cyclase activating polypeptide (PACAP), are linked to sepsis as their plasma expression rises during the early stages of sepsis disease models [8,9,10,11]. Moreover, due to their potent anti-inflammatory activities, these two peptides have been investigated in multiple sepsis models from multiple species [11,12,13,14,15].

VIP and PACAP are closely related pleiotropic neuropeptides of the secretin/glucagon peptide family that are distributed widely throughout the nervous, immune, and peripheral organ systems. They exert broad physiological effects, including neurotransmitter and neuromodulatory functions, neuroprotective activities, and regulation of cell growth and cell survival [16,17,18,19,20]. In sepsis-related settings, VIP and PACAP have attracted interest because of their potent immunoregulatory and anti-inflammatory properties, as well as their capacity to influence cytokine production, leukocyte activity, vascular tone, and tissue injury responses [8,9,10,11,12,13,14,15]. Their effects, however, are not uniformly protective and appear to depend strongly on experimental model, timing, tissue compartment, receptor context, and whether the principal outcome measured is inflammatory suppression, tissue protection, survival, or microbial clearance.

This review examines a central question that has emerged across four and a half decades of work, namely under what experimental conditions VIP and PACAP appear protective, and under what conditions they become permissive or potentially harmful in sepsis-related settings. We use a chronological framework not simply to recount the literature, but to show how this central tension emerged as the field moved from endotoxemia models to CLP-based polymicrobial injury, to live bacterial and viral infection models. Accordingly, this review focuses primarily on vasoactive intestinal peptide, while incorporating key studies on pituitary adenylate cyclase-activating polypeptide where they clarify mechanistic or conceptual parallels. The literature was assembled through targeted PubMed searches using the keywords vasoactive intestinal peptide and sepsis, which yielded 66 primary references forming the core historical dataset for this focused review. Additional papers were incorporated when necessary to clarify conceptual transitions, mechanistic advances, or points of tension in the field. Discoveries are summarized by decades, beginning with foundational observations in the 1980s and extending through studies of translational and mechanistic relevance in the 2020s. We highlight unresolved questions and future milestones for understanding when VIP and PACAP signaling is beneficial, when it is detrimental, and why.

Lastly, at each stage, we distinguish between endotoxemia, cecal-ligation and puncture (CLP), and live-infection models because these systems address overlapping but non-identical biological questions and therefore should not be interpreted interchangeably.

## 2. The Early Years: 1981–1989

This early phase of the literature was dominated by physiological association studies, and therefore primarily framed VIP as a correlate, and possible contributor, to lipopolysaccharide (LPS), an endotoxin found in outer membranes of Gram-negative bacteria (e.g., *Salmonella*), rather than as a defined immunoregulatory mediator.

After the discovery of vasoactive intestinal peptide in 1970 by researchers, Victor Mutt and Sami Said, seminal observations regarding the link between VIP and endotoxin shock were reported in the early 1980′s [9,10]. Freund H. et al. observed that VIP blood levels were peaked (≈250 pg/mL) after 2 h from LPS administration (1.5 mg/kg) into dogs that paralleled hypotension normally seen post-LPS injection. Surviving dogs 24 h post-endotoxin administration exhibited plasma VIP levels returning to baseline. Moreover, dogs that did not survive the LPS-induced shock had higher blood VIP levels compared to those that survived. This seminal finding suggested that VIP might act as a contributing factor in sepsis disease progression [10].

Similar findings were reported in pigs treated with *E. coli* LPS at 1 mg/kg of body weight. These porcine studies revealed that plasma VIP measured by radioimmunoassay became elevated as early as 30 min (0.11 nM) post-LPS injection and was maintained for at least 24 h with a slow decline over 60 h. There was a negative correlation between elevated plasma VIP levels and a drop in blood pressure with these early studies, and the source of elevated plasma VIP levels was thought to originate from the gastrointestinal tract as the rise in portal blood VIP levels was 3.7-fold higher than that measured systemically. In fact, surgically removing the gastrointestinal tract prior to endotoxin infusion resulted in no detectable rise in blood VIP [11].

Moreover, additional intestinal peptides were found increased in porcine endotoxic shock, including pancreatic polypeptide (PP), somatostatin, gastric inhibitory peptide (GIP) and insulin, which was subsequently confirmed to cause hypoglycemia during shock [11,21]. In 1986, researchers discovered that VIP levels rose from endotoxic shock with a positive correlation with endotoxin concentration and bacterial load. Noteworthy was that spikes in plasma VIP levels could be blocked during endotoxic shock if fluids were added to the animal simultaneously, which prevented the progression of shock [22].

**Figure 1:**
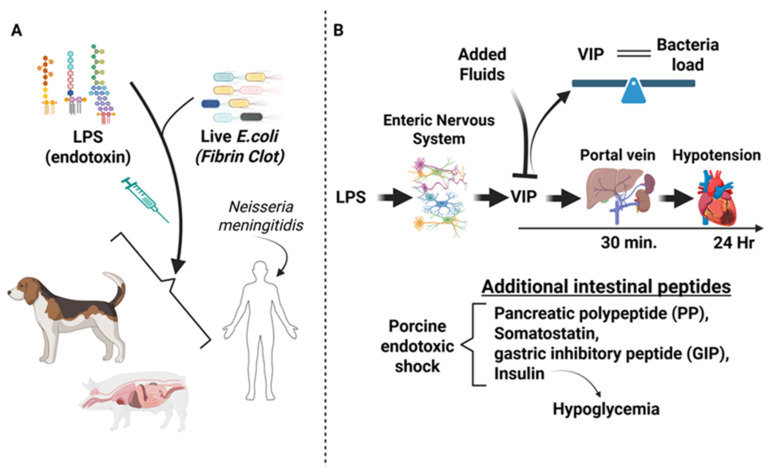
The Early Years; 1980–1989. (**A**). The 1980′s utilized LPS and live *E. coli*, sometimes from fibrin clots to administer to dogs and pigs for sepsis experimentation. Insights from humans suffering from *Neisseria meningitidis* were also used to add to the VIP experimental evidence of this decade. (**B**). This research provided evidence to suggest that LPS induced a rapid (30 min to 4 h) increase in VIP from neurons of the enteric nervous system of the gut, which peaked far earlier than hypotension (24 h). The horizontal arrow and time denote when VIP spikes in the portal vein and systemic hypotension were observed. If animals received fluids with endotoxin, VIP spikes were blocked. A positive correlation was found between VIP blood levels and bacteria load, and a balance is used to emphasize this equal correlation. Lastly, additional gut peptides were also identified as indicated, with insulin attributing to the observed hypoglycemic effects of shock.

The latter half of the 1980′s showed a wealth of additional data reported on the link between VIP and sepsis. With the added knowledge that VIP was expressed in the central and peripheral nervous systems and delivered to organs like the gastrointestinal tract, there was increased attention to understanding the mechanism for how VIP became elevated during sepsis and other hemodynamic circulatory collapse events, including trauma and hypovolemia [11]. Fuortes M. et al. made important advances with respect to the nature of VIP elevation in the blood induced by a fibrin clot containing live *E. coli* [23]. This study demonstrated that blood VIP elevation peaked at 4 h post sepsis induction (≈47 pM) in the portal vein versus Aorta (≈18 pM), which occurred far earlier than decreases in blood pressure that took place after 24 h. These results indicated that low blood pressure or hypotension was not the cause for VIP release during sepsis. In support of these animal studies, researchers also discovered that human patients suffering from meningococcal disease had elevated blood VIP (e.g., ≥4 pM) that correlated with high endotoxin levels (e.g., 200 ng/L). Antibiotic treatment lowered both blood LPS and VIP levels, suggesting that LPS stimulates the nervous system, likely the enteric nervous system in the gut, to release VIP (Figure 1) [24].

## 3. The Last Decade of the 20th Century

In this decade, the field began to move beyond descriptive peptide-release physiology and toward mechanism, particularly in endotoxemia models where VIP and PACAP emerged as suppressors of macrophage-derived inflammatory mediators.

### From Gut Peptide Release to Immune Modulation

In the early 1990s, research on neuropeptides increasingly focused on the gastrointestinal tract, particularly to understand pathophysiological changes induced by sepsis. Using a rat model of sepsis via cecal ligation and puncture (CLP), which leads to polymicrobial release of gut microbiota into the peritoneum, researchers measured various gut peptides via radioimmunoassay 16 h post-CLP and compared results to control animals. They observed elevated levels of gastrin (14.6 pg/mL), peptide YY (PYY; 108.4 pg/mL), secretin (127.2 pg/mL), and VIP (213.8 pg/mL), with the highest concentrations detected in portal blood rather than systemic circulation, suggesting that the gut was the primary source of these peptides. Interestingly, certain neuropeptides, such as gastrin-releasing peptide (GRP) and substance P, remained unchanged. Notably, tumor necrosis factor-α (TNF-α; 10 μg/mL—specific activity 5 × 10^6^ U/mg) was found to mimic CLP-induced upregulation of portal PYY, but did not affect VIP or other peptides, while interleukin-1α (IL-1α; 10 μg/mL—specific activity 3 × 10^8^ U/mg) failed to induce changes in any gut peptides. This study provided early mechanistic insights into CLP-induced neuropeptide regulation, particularly for PYY [25]. Two years later, Higashiguchi et al. showed that enterocytes isolated from septic (CLP) rats had increased total secreted protein synthesis and released more VIP (13.9 pg/10^6^ viable cells, 10–15—fold increase over control) and PYY (≈4.5 pg/10^6^ viable cells; 4—fold increase over control), with roughly half of the released peptides being newly synthesized as measured by ^3^H-phenylalanine incorporation. While the authors proposed that intestinal epithelial cells were the source of this synthesis and release, they acknowledged the possibility of neuronal origin contaminating their enterocyte system, as isolated epithelial cells contained low levels of nerve markers [26]. In 2025, VIP-reporter mice have confirmed that murine VIP expression within the submucosa is primarily expressed from enteric nerves innervating both the circular and longitudinal muscle layers and the submucosal plexus, the latter positioned in the submucosa that may have been the contaminating source of VIP from Higashiguchi’s study [27,28].

In New Zealand male rabbits, researchers examined the early “hyperdynamic/warm” phase of septic shock, induced by intravenous administration of a non-lethal dose of *E. coli* (2 × 10^8^ CFU/mL). They found that nitric oxide (NO) catalyzed by nitric oxide synthase (NOS) likely from activated blood phagocytes and other white blood cells mediated this early phase. The study demonstrated a rise in cell-associated cGMP, which correlated with key features of septic shock, including hypotension, bacteremia, elevated blood lactate, and acidosis. Administration of NG-Monomethyl-L-Arginine (L-NMMA), a competitive NOS inhibitor, blocked the rise in cGMP and nitrate (a proxy for NO production), confirming that NO-driven NOS activity in peripheral blood mononuclear cells (PBMCs) played a critical role in early septic shock [29]. Furthermore, this NO-induced septic shock acted synergistically with VIP by enhancing its release from neurons and prolonging NO signaling in target cells through increased intracellular cAMP, likely via phosphodiesterase inhibition. VIP and cAMP levels increased (≈1.4 ng/mL and 1.3 ng/mL, respectively) following NO and cGMP elevation, and although L-NMMA blunted their rise by about half depending on time post septic shock, they remained sufficient to sustain hypotension and lactate acidosis in later stages of septic shock. Collectively, these findings suggested that endotoxin-induced PBMC activation triggers NO–cGMP signaling in blood cells, vascular endothelial cells, and neurons, initiating early hyperdynamic septic shock. VIP released by enteric nerves [27] further amplifies this response via prolonged cAMP signaling, particularly by promoting smooth muscle vasodilation. This protective mechanism enhances blood flow to tissues, aiding PBMC-mediated clearance of endotoxins. However, excessive systemic activation of NO and VIP signaling can lead to life-threatening hypotension and circulatory collapse [29].

In rats, VIP administration (25 ng/kg) improved survival following lethal LPS exposure, although hypotension still occurred, albeit to a lesser extent than with LPS alone. Interestingly, inhibition of NOS with L-NAME (30 mg/kg), a non-selective NOS inhibitor, failed to reduce lethality, leading researchers to propose that VIP and NO may have opposing roles, while VIP administration improved survival, NOS inhibition had no protective effect [30]. Although the early VIP-sepsis literature relied mainly on non-selective NOS inhibition, later work in the broader sepsis field showed that selective manipulation of individual NOS isoforms yielded mixed and context-dependent results, with iNOS inhibition often improving hemodynamics, whereas nNOS- and eNOS-related effects were more variable and in some settings protective [31,32,33].

By the late 1980s, a structurally similar peptide to VIP, pituitary adenylate cyclase-activating polypeptide (PACAP), was discovered, sharing 68% amino acid homology with VIP [34,35]. Parallel studies demonstrated that both peptides exhibited potent anti-inflammatory properties by downregulating proinflammatory cytokine expression. Using a high-endotoxemia BALB/C mouse model (400 μg LPS from *Salmonella enteridis*), researchers found that 5 nmols of exogenous VIP or PACAP IP administration significantly reduced serum and peritoneal levels (≈60%) of TNF-α and IL-6 decreased lethality and mitigated associated histopathological damage. These findings established VIP and PACAP as protective agents against septic shock by attenuating TNF-α and IL-6 production. However, VIP alone failed to protect against TNF-α-induced septic shock, confirming that VIP’s protective mechanism operates upstream of TNF-α signaling [12]. Further mechanistic studies identified that 10^−8^ M VIP/PACAP-mediated downregulation of LPS-induced TNF-α expression was transmitted through VPAC1, a G protein-coupled receptor, in LPS-treated peritoneal macrophages [36,37,38,39]. VIP (10^−8^ M) binding to VPAC1 significantly suppressed TNF-α transcription in a cAMP-dependent manner. In vivo, VIP levels increased in serum and peritoneal fluid following LPS exposure, alongside proinflammatory cytokines such as TNF-α and IL-6 [12]. Subsequent studies led to the classification of VIP and PACAP as “macrophage deactivating factors [37].” Using in vitro models, researchers demonstrated that 10^−8^ M VIP and PACAP suppressed iNOS expression at both the mRNA and protein levels through cAMP-dependent and -independent pathways. This suppression was achieved by inhibiting interferon regulatory factor-1 (IRF-1) and NF-κB, respectively, with VPAC1 (and VPAC2 to a lesser extent) mediating the inhibition of iNOS expression [37]. Additionally, VIP/PACAP were shown to suppress IL-12, a key Th1-polarizing cytokine, through the VPAC1→cAMP/PKA pathway [36,38].

**Figure 2:**
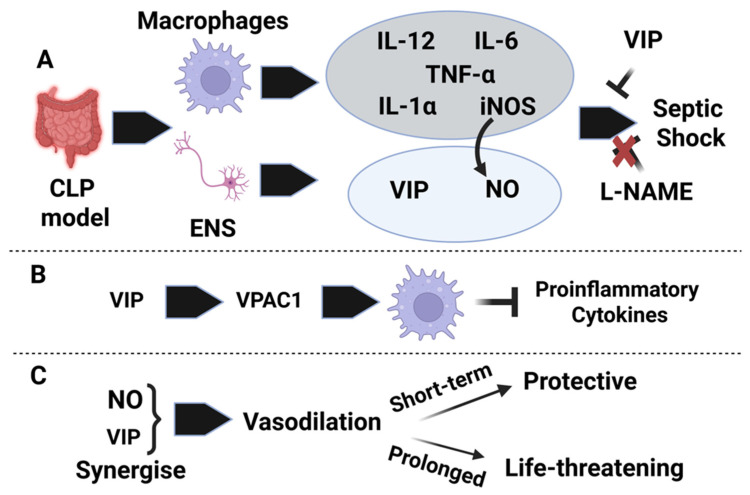
Conceptual summary of VIP, nitric oxide, and inflammatory signaling in sepsis studies from the last decade of the 20th century. (**A**) Early work in CLP models linked intestinal and enteric nervous system responses with macrophage activation, increased proinflammatory mediators, including IL-12, IL-6, TNF-α, IL-1α, and iNOS, and increased nitric oxide generation. Within this framework, VIP was associated with protection against septic shock, whereas nonselective NOS inhibition with L-NAME did not prevent lethality, highlighting a complex relationship between VIP and nitric oxide signaling in sepsis. (**B**) Mechanistic studies from this period also supported an anti-inflammatory role for VIP through VPAC1-dependent actions on macrophages that suppress proinflammatory cytokine production. (**C**) At the same time, VIP and nitric oxide could act in parallel to promote vasodilation, suggesting a dual effect in sepsis in which short-term vasodilatory responses may be protective, whereas prolonged signaling may become life-threatening by contributing to septic shock.

By the end of the decade, accumulating evidence confirmed the immunoregulatory roles of VIP and PACAP. These peptides effectively downregulated key proinflammatory mediators, including iNOS, IL-1, IL-6, IL-12, and TNF-α, in both in vitro and in vivo studies. Their potent ability to suppress macrophage activation, limit the immune response to LPS, and protect against endotoxin-induced lethality established VIP and PACAP as critical modulators of immune and inflammatory processes (Figure 2).

## 4. The First Decade of the 21st Century

This period consolidated the view of VIP and PACAP as broadly anti-inflammatory neuroimmune mediators, but it also introduced a growing need to distinguish systemic anti-inflammatory benefit from true antimicrobial benefit.

### VIP/PACAP Signaling Emerges as a Central Neuroimmune Regulatory Pathway

Vasoactive intestinal peptide (VIP) and pituitary adenylate cyclase-activating polypeptide (PACAP) are pleiotropic neuropeptides with strong immunomodulatory properties that operate primarily through three G-protein-coupled receptors (GPCRs): VPAC1, VPAC2, and PAC1. Both peptides bind VPAC1 and VPAC2 with equal affinity (1 nM kD), while PACAP selectively binds PAC1 (1 nM Kd) 1000-fold greater than VIP [40]. These receptors are differentially expressed across macrophages, dendritic cells, T cells, granulocytes, mast cells, and nonimmune tissues. The activation of these receptors suppresses proinflammatory cytokines such as TNFα, IL-1β, IL-6, IL-12, nitric oxide, and adhesion molecules like ICAM-1 and VCAM-1, while simultaneously upregulating IL-10, a major anti-inflammatory cytokine. In endotoxin (50 μg/mouse)-induced uveitis (EIU), where TNFα expression is heightened, VIP paradoxically exacerbated the ocular pathology. However, at higher LPS doses (up to 400 μg/mouse), co-administration of VIP (5 nmol/mouse) protected mice from death by suppressing systemic TNFα and IL-1β and increasing IL-10 [41]. These opposing effects illustrate the context-, dose, and compartment-dependent roles of VIP during systemic inflammation.

In LPS (10 mg/kg) and cecal ligation and puncture (CLP)-induced sepsis models in rats, VIP (5 nmol/rat for both studies) reduced proinflammatory cytokines (TNFα, IL-1β), elevated IL-10, reduced leukocyte infiltration, hemorrhage, ischemia, and necrosis in the intestine, and partially restored mean arterial pressure (MAP), consistent with improved hemodynamic stability, indicating systemic anti-inflammatory protection and improved tissue perfusion. VIP has a half-life of about 1 min in biological fluids like blood, which has limited its clinical translation [42]. However, encapsulation in sterically stabilized liposomes has improved peptide stability. Liposomal VIP stabilizes an α-helical conformation and prolongs peptide bioactivity, prevents rapid degradation, and increases half-life, making it a viable drug delivery strategy in sepsis and other inflammatory disorders [43,44]. VIP’s cellular targets include macrophages, dendritic cells, and mast cells. Delgado et al. (2000) showed that selective activation of VPAC1 and VPAC2 in LPS-challenged mice improves survival by suppressing TNFα, IL-6, IL-12, nitric oxide, and by increasing IL-10 [36]. Furthermore, VPAC1 downregulates B7.1/B7.2 co-stimulatory molecules, impairing antigen presentation and subsequent T cell activation. Gomariz et al. (2000) demonstrated that VIP and PACAP suppresses expression of CD14, inhibits transcription factors such as NF-κB, IRF-1, and c-Jun, while activating CREB-dependent transcription, thereby preventing overactivation of antigen-presenting cells [45,46]. These effects shift CD4+ T cell differentiation toward a Th2 phenotype, further suppressing Th1-driven inflammation.

Tuncel’s studies explored VIP’s effects on mast cells, revealing that VIP increases survival in septic rats without inducing oxidative stress in the liver or kidneys [47]. In the dura mater, LPS induced changes in mast cell subtype ratios: mucosal (MMC) and intermediate-type (IMC) cells increased, while connective tissue-type mast cells (CTMCs) decreased, along with elevated histamine and reduced serotonin levels. VIP potentiated degranulation in this brain region, while nitric oxide synthase (NOS) inhibition with L-NAME had no effect, suggesting a unique regulatory mechanism of VIP on CNS mast cells [48]. These mast cell effects may provide an additional layer of neuroimmune protection during septic shock. VIP suppresses Th1 polarization, which may attenuate antiviral immunity through reduced IL-12 and IFNγ production while simultaneously limiting inflammatory tissue injury.

From 2001 to 2006, several studies broadened the clinical implications of VIP/PACAP in inflammatory diseases beyond sepsis, including rheumatoid arthritis, Crohn’s disease, autoimmune diabetes, and multiple sclerosis [49,50]. Both neuropeptides reduce neutrophil infiltration, adhesion molecule expression (ICAM1, VCAM1), and fibrinogen synthesis—mitigating vascular injury and coagulopathy associated with septic shock [51]. PAC1-deficient mice displayed resistance to endotoxic shock due to a failure to induce IL-6 and other acute-phase proteins, emphasizing PAC1′s critical role in amplifying the inflammatory cascade. Importantly, VIP/PACAP functions are not fully reproduced by targeting a single receptor, and available evidence suggests that optimal anti-inflammatory protection involves coordinated signaling through VPAC1, VPAC2, and PAC1 [13,50,51,52]. VIP also modulates coagulation during sepsis. Tissue factor (TF) on monocytes is a central trigger of thrombin generation and thrombosis in septic patients. In vitro, VIP and PACAP downregulated TF at the mRNA, protein, and surface levels in human monocytes and THP-1 cells [53]. This effect was linked to inhibited NF-κB nuclear translocation and reduced phosphorylation of p38 and JNK MAPKs. While TF levels declined, tissue factor pathway inhibitor (TFPI) remained unaffected, demonstrating VIP’s selective suppression of procoagulant pathways without impairing regulatory components. Iqbal et al. proposed that many sepsis therapies fail by targeting only inflammation, instead of combining anti-inflammatory and antithrombotic therapies, such as suppression of TF expression and subsequent reduction in thrombin generation, which may be more effective [54,55].

VIP and PACAP form a communication bridge between the nervous and immune systems. Pozo (2003) emphasized their role as central mediators of the brain–immune axis, facilitating Th2 immune skewing and suppressing Th1-mediated disorders such as sepsis, inflammatory bowel disease, and autoimmunity [56]. Gonzalez-Rey’s review highlights VIP’s inhibition of proinflammatory cytokines and chemokines from macrophages, dendritic cells, and microglia, suppression of co-stimulatory molecules, and promotion of Th2-type immune profiles [57]. Waschek underscored their potential as alternatives to corticosteroids, given their systemic anti-inflammatory effects via high-affinity GPCRs and favorable safety profiles in various animal models [58]. Evidence from knockout studies supports these conclusions. VIP KO mice exhibit increased airway reactivity to methacholine, heightened perivascular and peribronchial inflammation, and higher mortality from endotoxemia. Interestingly, male KO mice died earlier than wild-type but later than female KO mice, indicating a sex-specific role of VIP in host survival [59]. In clinical models of peritonitis, patients demonstrated reduced substance P (SP; 80% reduction) and elevated 2-fold VIP in intestinal tissues (5.1 ± 1 vs. 10.2 ± 0.55% immunoreactivity within the myenteric plexus). Since VIP slows intestinal transit and SP is prokinetic, this inverse correlation may explain delayed motility observed during peritoneal inflammation. Although not mechanistically dissected, these data suggest that altered neuropeptide concentrations influence gut function in sepsis [60]. In contrast, VIP (7.3–10.9 pmol/mL) was not altered in an LPS model (2 ng/kg) mimicking bacterial meningitis, while CGRP increased and ET-1 was abolished. The absence of VIP modulation in this context may reflect compartmental specificity or a divergence in neuropeptide regulation in CNS infections. Finally, cytokine expression dynamics revealed that TNFα peaks at 2 h post-LPS exposure, while IL-1 and IL-10 rise later, suggesting that the timing of VIP administration may be critical for achieving maximal therapeutic benefit [61].

**Figure 3:**
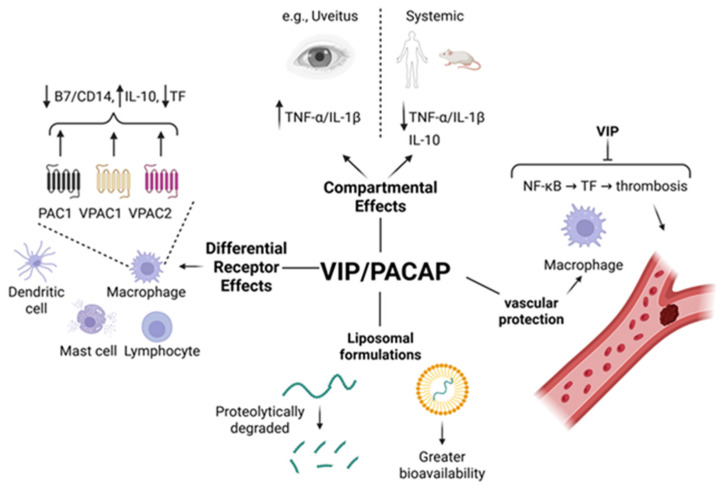
Multifaceted Roles of Vasoactive Intestinal Peptide (VIP) in Immune Regulation and Therapeutic Potential. This schematic illustrates the context-dependent, receptor-specific, and cell-type targeted effects of VIP, as well as strategies to enhance its therapeutic stability. They include (1) Dual Nature in Inflammation, (2) Receptor-Specific Modulation, (3) Immune Cell Targets, (4) Coagulation Control, and (5) Delivery and Stability.

Emerging evidence suggests that VIP signaling functions not simply as an anti-inf [60] lammatory pathway but as a regulator of tissue homeostasis integrating neural, metabolic, and immune signals. Defining the epithelial and metabolic targets of VIP signaling may therefore reveal new therapeutic approaches for inflammatory and metabolic diseases (Figure 3).

## 5. The Teenage Years

By this stage, the field had begun to reveal a central tension, namely that VIP could suppress damaging inflammation and tissue injury while simultaneously weakening microbial clearance in the setting of live infection.

### VIP/PACAP as Regulators of Immune Cell Responsiveness and Host–Pathogen Balance

While the first decade of the 21st century recognized VIP and PACAP as potent silencers of the cytokine storm, the teenage years (i.e., from 2010 to 2019) revealed that these neuropeptides also regulate which immune cells enter tissues, and when, by modulating upstream sensing, adhesion, and chemotactic pathways. This work redefined VIP and PACAP as regulators not only of cytokine production but also of immune cell responsiveness and trafficking during sepsis.

In rat models of LPS-induced acute lung injury, VIP reduced inflammatory cell infiltration into the alveolar spaces, preserved lung architecture, and limited protein leakage, effects mechanistically linked to downregulation of TLR2 and TLR4 expression. This in turn dampened local production of chemokines and adhesion molecules that drive leukocyte extravasation [62]. Complementary work showed that VIP re-balances the TREM-1/TREM-2 ratio in lung macrophages, shifting these cells toward an anti-inflammatory, tissue-resident phenotype rather than a pro-inflammatory, “recruit-more-cells” state [63]. In a separate rat model study, intravenous LPS induced interstitial edema, inflammatory cell infiltration, and destruction of the inter-alveolar space of the lungs. VIP treatment (5 nmole/kg, alone or in combination with glucocorticoids (GC, 3 mg/kg), attenuated this lung damage. LPS (10 mg/kg) downregulated glucocorticoid receptor (GR) mRNA, but VIP partially preserved this expression, and VIP + GC fully restored GR expression, suggesting that enhanced GC→GR signaling may underlie the observed protection [64].

Paradoxically, global VIP knockout mice were highly resistant to LPS-induced endotoxemia. Intraperitoneal (IP) administration of LPS into VIP-deficient mice resulted in lower mortality and reduced tissue damage. Closer examination revealed reduced proinflammatory cytokine expression (e.g., IL-6, IL-12, and TNFα) in peritoneal cells, the spleen, and the lung, supporting the idea that VIP deficiency results in an abnormally reduced ability of innate immune cells to sense and become activated by LPS, perhaps through reduced levels or activity of TLR2/4, as described by Zuo two years earlier. In support of this notion, the authors provided evidence that VIP deficiency resulted in suppressed activation of the NF-κB pathway downstream of TLR2/4 [65]. These genetic studies utilizing global VIP KO mice, which exhibit responses to LPS similar to those observed in WT mice treated with exogenous VIP, suggest that this “intrinsic defect,” as the authors described it, could reflect an adaptation of immune cell signaling that develops in the absence of VIP. A scientific precedent for this idea is the effect of commensal gut bacteria on innate immune development. Commensal microbes normally induce innate immune activation and proinflammatory cytokine secretion, but if the host develops in the absence of a microbiota (e.g., germ-free mice), the immune system is underdeveloped and hyporesponsive as a result [66]. Taken together, VIP functions both as an acute anti-inflammatory mediator and, possibly, as a developmental regulator of innate immune responsiveness.

**Figure 4:**
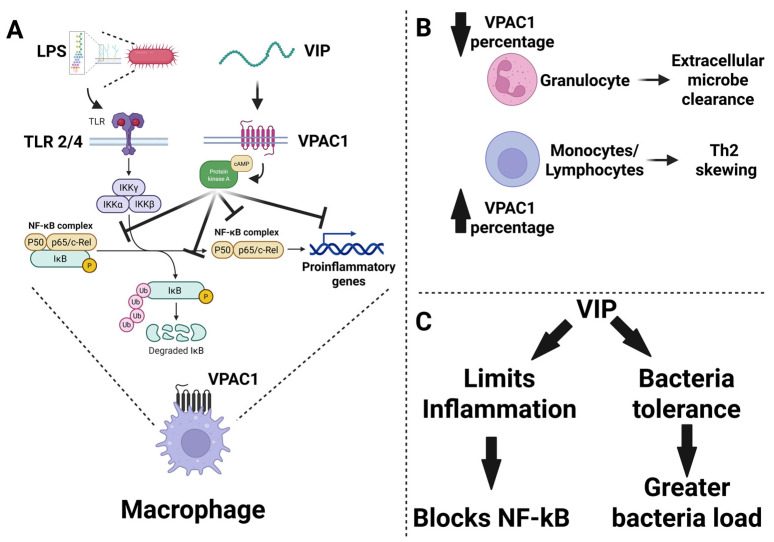
Conceptual summary of VIP signaling through VPAC1 during sepsis and infection-related inflammation. (**A**) Lipopolysaccharide, LPS, derived from Gram-negative bacteria activates Toll-like receptor 2 and 4 signaling and promotes IKK-dependent phosphorylation and degradation of IκB, allowing NF-κB complexes to drive transcription of proinflammatory genes in macrophages. In parallel, VIP signals through VPAC1, engaging cAMP and protein kinase A pathways that interfere with NF-κB activation and transcriptional output, thereby suppressing macrophage inflammatory responses. (**B**) Proposed shifts in VPAC1-associated immune balance are shown schematically, with reduced VPAC1 representation in granulocytes linked to impaired extracellular microbe clearance and increased VPAC1 representation in monocytes and lymphocytes associated with Th2 skewing. (**C**) Together, these studies support a dual model in which VIP can limit inflammation through blockade of NF-κB signaling, yet under some infectious conditions this same immunoregulatory effect may promote bacterial tolerance and contribute to greater microbial burden.

Additional evidence for a neuroendocrine regulatory loop upstream of VIP expression came from adrenal studies, where systemic LPS increased adrenal VIP and galanin mRNA levels. This effect was dependent on PACAP signaling, as PACAP KO mice failed to upregulate either peptide. In vitro, 100 nM PACAP plus 10 nM TNF-α induced long-term expression of VIP and galanin in chromaffin cells, potentially by reducing IkBα-mediated inhibition of NF-κBα and thereby prolonging TNF-α-induced transcriptional activity [67]. This work highlights PACAP’s upstream control of VIP expression in the adrenal stress response. In a pig model of fecal peritonitis-induced sepsis, VIP levels were significantly elevated in plasma and mesenteric arteries (≈1 ng/g), while coronary artery levels were depleted by 75% possibly due to depleted neuronal content as suggested by the authors [68]. These data suggest a regional redistribution of VIP toward the splanchnic circulation, perhaps reflecting prioritization of anti-inflammatory tone in organs vulnerable to permeability and microbial translocation, like the gut (Figure 4).

VIP’s immunoregulatory effects are primarily mediated through the VPAC1 receptor, which is expressed in the immune system on granulocytes, monocytes and lymphocytes. In a human endotoxemia model, LPS administration (2 ng/kg) caused dynamic changes in VPAC1 expression across immune cell subsets. Granulocytes, accounting for half of the leukocytes, expanded 2.6-fold after LPS, but the proportion expressing VPAC1 dropped sharply at 3 h (58% vs. 28%) and returned to baseline by 24 h. Conversely, LPS increased the percentage of monocytes and lymphocytes expressing surface VPAC1 over 24 h (11% and 6% vs. 31.6% and 13.2%, respectively). Plasma VIP levels rose modestly and transiently (0.5 ng/mL vs. 0.7 ng/mL). These data suggest a dynamic redistribution and regulation of VPAC1 expression during endotoxemia, which may influence cellular responsiveness to VIP during inflammation [69]. VIP also acts on dendritic cells (DCs) to induce a tolerogenic phenotype. Lentiviral transduction of DCs with VIP cDNA created VIP-secreting DCs that failed to upregulate costimulatory molecules and inflammatory cytokines after CLP-induced sepsis. Instead, they promoted IL-10 production and likely induced regulatory Tregs, offering a cell-based alternative to systemic VIP administration, which is limited by rapid degradation and systemic effects [15].

In parasitic infection, every other day IP injections of 1.5 nmol/animal of VIP dampened inflammation in Trypanosoma cruzi-infected mice (5000 trypomastigote forms), shifting the cytokine profile toward IL-4 and IL-10 while reducing IFN-γ and cardiac pathology, without significantly altering parasite burden [70]. Similarly, in rats with sepsis-induced encephalopathy, VIP treatment reduced cognitive dysfunction by blocking TLR4/NF-κB signaling in the hippocampus, decreasing neuronal apoptosis and proinflammatory cytokines. These findings highlight that VIP is not just a macrophage-deactivating factor but a broad regulator of proinflammatory signaling cascades across multiple cell types and organs [71].

Although VIP has demonstrated strong anti-inflammatory and tissue-protective effects in models of endotoxemia, its role in the context of live bacterial infection is more nuanced and, in some cases, problematic. In *Salmonella typhimurium*-infected monocytes (multiplicity of infection (MOI), 10:1), 10 nM of VIP exerted differential effects depending on the microbial stimulus. While 100 ng/mL LPS or live *Salmonella* both induced inflammatory cytokines (TNF-α, IL-1β, IL-6), VIP co-treatment blunted the response more effectively in LPS-treated monocytes than in live bacterial infection, likely due to the complexity of multiple microbe-associated molecular patterns (MAMPs) present in viable pathogens [72]. Despite increasing monocyte viability, VIP also permitted higher bacterial loads at 6 and 24 h post-infection, indicating a tradeoff between immunosuppression and pathogen control.

In murine models, 5 nmols of VIP IP treatment increased *Salmonella* burden (MOI 10:1) in the gut, mesenteric lymph nodes (MLNs), and spleen while reducing inflammatory cytokines. These results suggest that VIP promotes immune tolerance or suppression, possibly to maintain host-microbiota homeostasis, but at the cost of impaired pathogen clearance [73]. This anti-inflammatory effect is consistent with VIP’s effects on shifting from type I (anti-bacterial) to type II (anti-worm) immunity recently reported in 2025 [27,28]. In support of this, *Salmonella* infection induced VPAC1 mRNA expression in ileum, MLNs, and spleen, and the presence of VIP enhanced bacterial colonization, suggesting a VIP→VPAC1 signaling pathway regulating peripheral immune tolerance. The authors concluded that while VIP mitigates inflammatory damage, it may enable microbial persistence, thus highlighting a cautionary consideration for its use in treating polymicrobial sepsis. Additional work shows that *Salmonella* may exploit VIP→VPAC1 signaling to evade host immunity. In infected monocytes, *Salmonella* (MOI 10:1) induced VPAC1 surface expression and recycling, increasing receptor availability. VIP co-treatment (10 nM) further increased intracellular bacterial burden. Calmodulin 1 appeared to mediate VPAC1 trafficking, suggesting *Salmonella* manipulates host GPCR trafficking pathways to enhance intracellular survival [74]. At the signaling level, 10 nM VIP modulated JAK/STAT pathways in *Salmonella*-infected monocytes (MOI, 10:1). While *Salmonella* upregulated IFNGR1 and IL-6R, VIP blocked IFNGR1 protein but not mRNA, implying interference with receptor trafficking. VIP also suppressed key cytokines (IFNγ, IL-20), increased SOCS1/3, and enhanced ISG15, a gene associated with increased susceptibility of Gram-negative bacteria infection. This raises concerns about the immunosuppressive ceiling of VIP treatment in live infections [75].

Given VIP’s rapid degradation and systemic effects, efforts have turned toward developing stable analogs with enhanced therapeutic potential and improved antimicrobial activity. Campos-Salinas et al. (2014) synthesized two VIP synthetic analogs, VIP51 and VIP51(6–30), that retained anti-inflammatory properties while gaining potent antimicrobial activity due to increased cationic charge [76]. The first analog, VIP51, was 30 amino acids long with two extra arginine residues at amino acid position 29 and 30, with amino acid substitutions at positions 8 (D→A), 17 (M→L), 24 (N→S), 25 (S→A), and 28 (N→G). The second analog, VIP51(6–30), was identical to VIP51 except the absence of the first 5 amino acids at the N-terminus (e.g., amino acids 6–30). These analogs disrupted microbial membranes by forming pores, killing both pathogenic and non-pathogenic bacteria as well as *Leishmania major*. Importantly, they did not affect eukaryotic cells. In a polymicrobial sepsis model, both analogs lowered bacterial burden and inflammation, and VIP51(6–30) reduced lesion size in a leishmaniasis model. These results suggest that rational design of VIP-based therapeutics may allow optimization of the balance between inflammation suppression and microbial clearance.

Parallel approaches in Sprague-Dawley rats using radical scavengers and cytokine inhibitors have also shown promise. Schulz et al. (2015) demonstrated that parecoxib (a COX-2 inhibitor) and IP administered VIP (10 ug/kg) offered protection against LPS-induced (1 mg/kg IP injection) intestinal barrier permeability in the large intestine, while pyruvate and Tempol were more effective in the small intestine [77]. This regional specificity suggests synergistic therapeutic combinations targeting distinct gastrointestinal zones during sepsis. Glucagon-like peptide-2 (GLP-2), another gut hormone, has also demonstrated barrier-protective effects in endotoxemia [78].

Expanding beyond VIP alone, related neuropeptides such as PACAP and their shared receptors (VPAC1, VPAC2, and PAC1) contribute to coordinated endocrine-immune responses during infection and inflammation. In a comparative study, rainbow trout were exposed for 4 h at 12 °C to a highly pathogenic isolate of viral haemorrhagic septicaemia virus at a dose of 5.56 × 10^5^ infectious units/mL. PACAP, VIP, and their receptors were differentially expressed in primary lymphopoietic organs (kidney and spleen). Splicing variants of PACAP and its receptors were altered in response to infection, indicating dynamic regulation at the transcriptional and post-transcriptional levels [79]. This cross-talk between neuropeptides and immune signaling appears to be evolutionarily conserved and crucial for maintaining immune homeostasis during systemic infection. Finally, broader neuromodulation strategies are emerging. CXCR1/2 inhibitors, such as Reparixin, improved lung histology in LPS-induced sepsis and modulated neuropeptide expression, notably increasing substance P and opiomelanocortin [80]. Though not directly tied to VIP, this suggests that targeting neuropeptide-receptor systems broadly, including VIP/PACAP and related pathways, may offer novel entry points for treating endotoxic shock and modulating immune tone in a tissue-specific manner.

**Figure 5:**
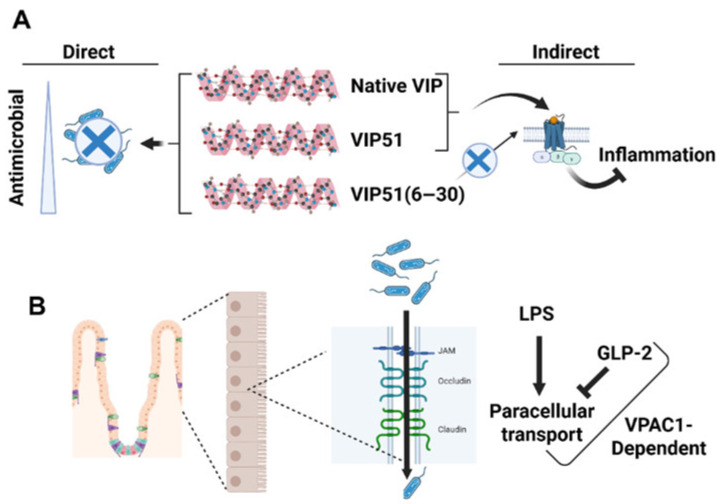
Engineered VIP Analogues Exhibit Dual Anti-Inflammatory and Antimicrobial Functions and Interact with Gut Barrier Signaling. (**A**) Synthetic VIP analogues such as VIP51(6–30) demonstrate dual functionality by directly disrupting microbial membranes (antimicrobial effect) while improving bactericidal properties by an order of magnitude compared to native VIP. Both analogues are structurally optimized to enhance pathogen-killing capacity without compromising anti-inflammatory signaling. Visual representation includes the three peptides and their direct and indirect actions. (**B**) VIP signaling through VPAC1 interacts with gut barrier function and synergizes with GLP-2 to reduce intestinal permeability following LPS challenge. A cross-sectional schematic of the intestinal epithelium illustrates tight junction preservation mediated by VIP and GLP-2, with VPAC1 serving as a central signaling node in the gut-immune-neuroendocrine axis.

To summarize the major findings of this decade, these studies position VIP and related neuropeptides as central components of a neuroendocrine–immune network that integrates inflammatory signaling, epithelial barrier function, and host defense during sepsis. VIP signaling consistently limits inflammatory tissue injury and preserves barrier integrity, yet in the setting of live infection this same anti-inflammatory activity may compromise microbial clearance. This duality suggests that successful therapeutic strategies will likely require context-dependent modulation of VIP→VPAC signaling rather than simple augmentation or inhibition. Future work defining cell-type specific receptor signaling, temporal dynamics of VIP expression, and interactions with microbial stimuli will be essential for translating VIP-based approaches into effective therapies for sepsis and endotoxemia (Figure 5).

## 6. The Roaring Twenties

Recent studies have sharpened the distinction between barrier-protective and anti-inflammatory effects on one hand, and infection-permissive consequences on the other, bringing the translational limits of generalized VIP augmentation into much clearer focus.

### Context-Dependent Roles of VIP in Host Defense and Neuroendocrine–Immune Integration

Although fewer in number, recent studies in the 2020s have sharpened our understanding of VIP’s role in sepsis, particularly its nuanced effects on infection dynamics, gut barrier integrity, neuroinflammation, and broader endocrine-immune interactions. Askar et al. (2020) not only confirmed the protective role of VIP in endotoxemia, but they also pushed the field forward by evaluating VIP’s effects during live Gram-negative bacterial infection [73]. Using *Salmonella typhimurium* 4/74, the authors noted increased VPAC1 mRNA expression in the ileum and mesenteric lymph nodes by day 6 post-infection, and in the spleen as early as day 3. VIP administration led to an increased burden of *Salmonella* in the gut, lymph nodes, and spleen, while suppressing the release of inflammatory cytokines. These findings suggest that while VIP suppresses classical sepsis biomarkers like TNFα and IL-6, it may do so at the expense of immune clearance. The authors conclude that VIP may create conditions that favor microbial persistence through immunosuppressive effects, potentially reflecting its broader role in gut immune tolerance and microbiota-host coexistence [81,82]. Importantly, they propose that VIP can be a valuable adjunctive therapeutic, particularly when excessive inflammation, rather than microbial burden, is the key driver of pathology.

Maruta et al. (2020) investigated gut barrier dysfunction as a key contributor to endotoxin-induced pathology [78]. Using FITC-dextran to measure paracellular flux, LPS administration increased intestinal permeability within six hours, accompanied by elevations in endogenous GLP-2, proglucagon expression, and inflammatory cytokines [78]. Treatment with exogenous GLP-2 reversed these effects, reducing FITC-dextran levels in the portal circulation and suppressing cytokine expression, demonstrating a clear barrier-protective and anti-inflammatory effect in the small intestine. Importantly, the protective actions of GLP-2 were abolished by the VPAC1 antagonist PG97-269 and by the nitric oxide synthase inhibitor L-NAME, but not by inhibition of EGF or IGF-1 signaling, indicating a VIP→VPAC1- and NO-dependent mechanism. These findings reveal a previously unrecognized interaction between GLP-2 and VIP signaling in the regulation of epithelial barrier integrity during endotoxemia. The requirement for intact VPAC1 signaling suggests that GLP-2–mediated barrier stabilization may depend on endogenous VIP activity. Together, these observations suggest that GLP-2 and VIP signaling pathways may act cooperatively to preserve gut integrity during endotoxin exposure and raise the possibility that adjunctive therapies combining GLP-2 with intact or enhanced VIP signaling could improve barrier protection while limiting inflammatory injury.

Yang et al. (2022) extended VIP’s protective portfolio to the central nervous system [71]. In a CLP-induced model of sepsis-associated encephalopathy (SAE), VIP expression was significantly reduced by 75% in the hippocampus (relative expression at mRNA and protein level). VIP supplementation by stereotactic injection of adeno-associated virus -VIP attenuated cognitive dysfunction by suppressing TLR4/NF-κB signaling, reducing neuronal apoptosis, and lowering hippocampal inflammatory cytokines. These results support the concept that VIP is not only a macrophage-deactivating factor, but also a neuroprotective agent in the specific context of sepsis-associated encephalopathy, capable of modulating glial and neuronal responses to systemic inflammation.

**Figure 6:**
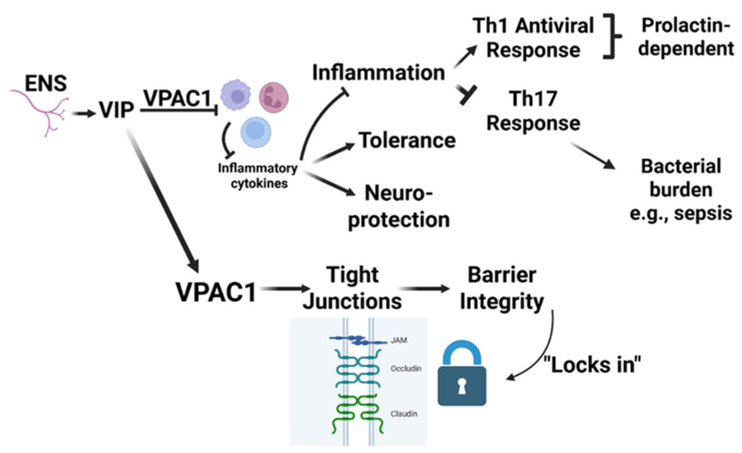
VIP-VPAC1 signaling in gut barrier and immune regulation. This schematic illustrates the role of vasoactive intestinal peptide (VIP), released from the enteric nervous system (ENS), in maintaining intestinal homeostasis through its interaction with the VPAC1 receptor. VIP binding to VPAC1 suppresses inflammatory cytokine production, promoting anti-inflammatory effects including immune tolerance and neuroprotection. This pathway downregulates Th1 and Th17 responses, the latter of which is associated with increased bacterial burden and sepsis risk. Th1 and Th17 responses are shown to be prolactin-dependent. Simultaneously, VPAC1 signaling enhances expression and maintenance of tight junction proteins (e.g., JAM, occludin, claudin), reinforcing epithelial barrier integrity. The resulting strengthened barrier “locks in” protection against pathogen invasion and inflammation, further contributing to intestinal immune homeostasis.

Mo et al. (2021) provided indirect evidence for VIP involvement in antiviral immune regulation using a chicken model of avian leukosis virus subgroup J (ALV-J) infection [83]. Circulating prolactin levels declined during infection, whereas exogenous recombinant prolactin suppressed viral replication and reduced proinflammatory cytokine production. This antiviral and anti-inflammatory effect was accompanied by increased VIP expression (relative mRNA increase), suggesting a functional association between prolactin signaling and VIP regulation. These observations point to a potential prolactin→VIP regulatory axis linking endocrine signals with antiviral immunity, although the mechanistic basis remains unclear and the evidence is largely correlative. Prolactin is generally considered a Th1-promoting immunomodulatory hormone, yet in viral infection models it can reduce excessive inflammatory cytokine production, possibly through secondary mediators such as VIP. Together, these findings suggest that prolactin and VIP may act cooperatively to balance antiviral defense with control of inflammatory damage, raising the possibility of a coordinated neuroendocrine feedback mechanism during viral infection (Figure 6).

## 7. Conclusions

A consistent message across this literature is that VIP and PACAP are not uniformly protective or harmful; rather, their effects depend heavily on whether the experimental system models sterile endotoxemia inflammation, polymicrobial injury, or live infection in which pathogen clearance must be preserved. Over the past four and a half decades, research on the neuropeptides VIP and PACAP in the context of sepsis has evolved from early physiological observations of peptide release during endotoxemia to mechanistic and systems-level investigations revealing their central roles in immune regulation, barrier protection, and neuroendocrine control of inflammatory responses. This historical arc has revealed that VIP exhibits both pro- and anti-inflammatory molecular and cellular effects, contingent on dose, receptor subtype activation, temporal expression patterns, and the immunological milieu. This dual nature has complicated the translation of preclinical findings into clinical therapies but also underscores the importance of VIP signaling in fine-tuning immune responses. A central challenge in advancing neuropeptide-based therapeutics for sepsis is the complexity of the disease itself. For instance, the heterogeneity in host response, timing of intervention, and variable microbiological triggers make it difficult to identify a one-size-fits-all approach. Furthermore, the pleiotropic effects of VIP and PACAP raise concerns regarding off-target consequences, such as hypotension or immunosuppression, not to mention the gastrointestinal tract, especially in critically ill patients. Therefore, conditional KO mice may offer a key strategic research tool to gain a better understanding of VIP signaling through each of its cognate receptors, VPAC1 and VPAC2. Moreover, future studies must refine our understanding of VIP and PACAP signaling in sepsis by incorporating systems biology approaches, including spatial and temporal mapping of receptor expression, transcriptomic analyses in immune and parenchymal cells, and patient stratification based on immunophenotypes. Importantly, novel delivery methods, such as targeted nanoparticles or receptor-selective analogs, may allow for greater therapeutic precision and fewer adverse effects.

VIP has demonstrated the ability to modulate cytokine storms, preserve epithelial barrier integrity, and prevent organ dysfunction, which are hallmarks of severe sepsis and septic shock. Therefore, the therapeutic potential of VIP and neuropeptides in sepsis remains promising. However, several key challenges remain, including identifying the optimal dose, refining drug delivery strategies, and determining the most effective timing of intervention during the progression of sepsis. Future studies should also incorporate important biological variables such as age and sex and expand investigations to include septic patient populations in order to better translate findings from animal models to human disease. As research continues to bridge gaps between animal models and human disease, neuropeptides and their downstream signaling targets may emerge not only as adjunct therapies but also as important components of precision immunotherapy strategies designed to modulate immune responses while preserving antimicrobial host defenses. Ultimately, defining the spatial and temporal dynamics of VIP (and PACAP) signaling across immune, epithelial, and neural compartments may provide new opportunities to harness neuropeptide biology for the treatment of sepsis and other inflammatory disorders.

## Data Availability

Data sharing is not applicable. No new data were created or analyzed in this study.
